# Estimating the effects of transcription factors binding and histone modifications on gene expression levels in human cells

**DOI:** 10.18632/oncotarget.16988

**Published:** 2017-04-09

**Authors:** Lu-Qiang Zhang, Qian-Zhong Li

**Affiliations:** ^1^ Laboratory of Theoretical Biophysics, School of Physical Science and Technology, Inner Mongolia University, Hohhot, China

**Keywords:** transcription factors, histone modifications, DNase-I hypersensitivity, statistical redundancy, regulation mechanism

## Abstract

Transcription factors and histone modifications are vital for the regulation of gene expression. Hence, to estimate the effects of transcription factors binding and histone modifications on gene expression, we construct a statistical model for the genome-wide 15 transcription factors binding data, 10 histone modifications profiles and DNase-I hypersensitivity data in three mammalian. Remarkably, our results show POLR2A and H3K36me3 can highly and consistently predict gene expression in three cell lines. And H3K4me3, H3K27me3 and H3K9ac are more reliable predictors than other histone modifications in human embryonic stem cells. Moreover, genome-wide statistical redundancies exist within and between transcription factors and histone modifications, and these phenomena may be caused by the regulation mechanism. In further study, we find that even though transcription factors and histone modifications offer similar effects on expression levels of genome-wide genes, the effects of transcription factors and histone modifications on predictive abilities are different for genes in independent biological processes.

## INTRODUCTION

Earlier studies [1–4] showed transcription factors (TFs) binding and histone modifications (HMs) were critical for gene expression, and the abnormities of TFs binding and HMs may affect the cell fate such as differentiation and apoptosis [5]. The ability to comprehend and predict their effects is vital to develop treatments for hundreds of human diseases, including leukemia [6], diabetes [7] and various cancers such as prostate cancer [8, 9], lung cancer [10] and breast cancer [11, 12], etc.

The significant regulations of mammalian gene expression are deemed to occur at the level of transcriptional initiation and elongation [13]. TFs can activate or block the initiation of gene transcription by binding to specific DNA sequences in enhancers or promoters [14, 15] or recruiting some chromatin-modifying enzymes to induce the changes of chromatin structure [16]. HMs are recognized to activate or inhibit transcription by either modulating the local chromatin structure to control TFs accessibility [17] or directly recruiting related enzymes [18].

In previous studies, by analyzing the relations of HMs and TF binding to gene expression, Cheng et al. [19] found that HMs or TFs binding in different positions show different predictive abilities, and they suggested HMs and TF binding may be redundant for predicting gene expression levels. Karlic et al. [20] noticed that different combinations of HMs are needed for predicting the expression levels of genes with different CpG content promoters. In this study, we investigate the relative contribution of each TF (HM) or combination of them to gene expression by constructing a support vector regression (SVR) model for the genome-wide 15 TFs binding data, 10 HMs profiles and DNase-I hypersensitivity data in three mammalian, and verify their universality in H1-HESc, Gm12878 and K562 cell lines. We further explore how TFs, HMs and gene expression interact with each other. At last, we research the effects of TFs and HMs on prediction for genes in independent biological processes.

## RESULTS AND DISCUSSIONS

### The “Optimal” TFs for predicting gene expression are cell-specific

**Figure 1:**
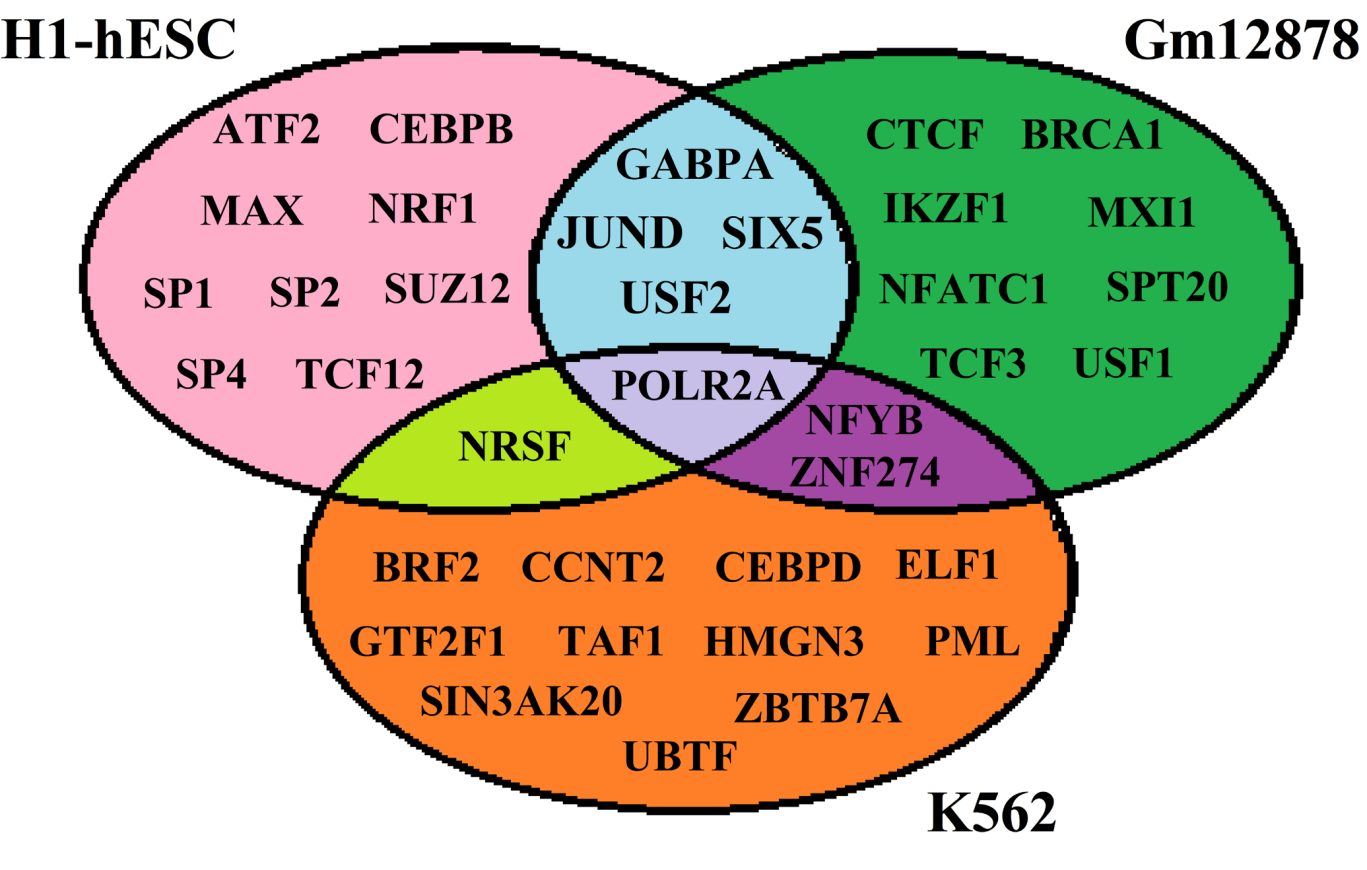
List of the TFs involved in the current study for H1, Gm12878 and K562

TFs can bind to specific DNA elements and stimulate or suppress gene transcription. There are approximately 1700 to 1900 TFs in human, including 1391 manually curated sequence-specific TFs [5]. In this study, we download respectively available 57, 87 and 96 TFs for H1-hESc (human embryonic stem cells), Gm12878 (B-lymphoblastoid cell) and K562 (erythrocytic leukemia cells) which are immortal [21] and have the most completed data [22]. Then the top 15 TFs which are vital TFs for predicting gene expression levels are chosen by using stepwise regression analysis (the usage about stepwise regression analysis is detailed in Supplementary information), and regarded as the “optimal” TFs for each cell line (shown in Figure 1). We observe that different “optimal” TFs are needed for different cell lines, indicating TFs binding is a dynamical process that depends on tissues or cell lines. A likely explanation for these phenomena may be the essential difference among the three cell lines, necessitating the selection of alternative TFs [2].

### TFs and HMs predict gene expression levels

The presences or absences of some TFs and HMs are correlated with gene expression levels [1, 16, 20, 23]. To better understand the relations between TFs (HMs) and gene expression levels, we construct log-linear model and non-linear SVR model for three immortalized human cell lines: H1-hESC, GM12878 and K562. The predictive power (*R*^2^) of the two models in 10-fold cross-validation are shown in Table 1 and Supplementary file Table S1.

**Table 1:** Prediction accuracy of log-linear and SVR model

		TFs	HMs+DNase	TFs+HMs+DNase
**H1**	log-line regression	0.404	0.529	0.555
	SVR	0.544	0.594	0.635
**Gm12878**	log-line regression	0.495	0.668	0.649
	SVR	0.617	0.719	0.730
**K562**	log-line regression	0.527	0.641	0.633
	SVR	0.627	0.690	0.688

The *CV-R*^2^ is the average R^2^ for the 10 fold cross-validation.

The results show that TFs, HMs and DNase have stronger correlation with gene expression levels in SVR model than in log-linear model. It may be resulted from the non-linear relationships between TFs (HMs) and gene expression [19, 24]. Therefore, SVR model is applied in the remainder of this work, despite a remarkable increase in required CPU time.

### Different HMs and TFs are required for predicting gene expression levels

**Figure 2:**
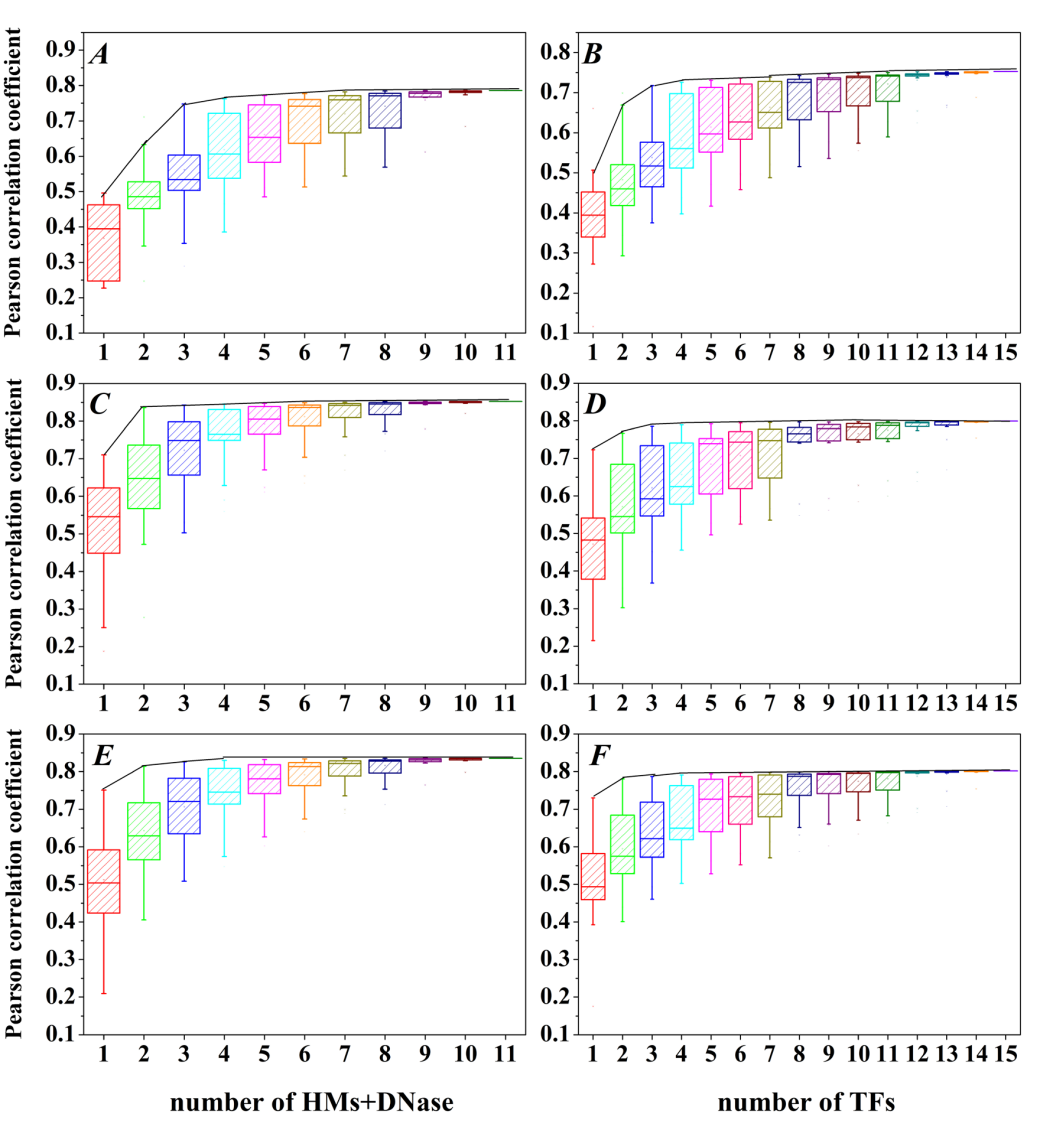
The PCC distributions for all combination of 15 TFs or 10 HMs and DNase **A**., **B**. H1, **C**., **D**. Gm12878 and **E**., **F**. K562 cell line. X-axis represents the combination of c kinds of HMs and DNase (choose c out of 10 HMs and DNase, c = 1,2,…,11) or d kinds of TFs (choose d out of 15 TFs, d = 1,2,…,15), and the black curves represent the maximum PCC for the combination mode of c HMs and DNase or the combination mode of d TFs.

In order to check whether all HMs (TFs) are equally important for predicting gene expression, we construct SVR models for all possible combinations of 10 HMs and DNase or 15 TFs, which results in 2047 HMs+DNase combination modes and 32767 TFs combination modes. The detailed information and statistical results are depicted in Supplementary tables 1-6 and Figure 2. The distributions of Pearson correlation coefficient (PCC) for these 2047 HMs+DNase combination modes in the H1-hESc, GM12878 and K562 are respectively shown in Figure 2A, 2C and 2E). The distributions of PCC for these 32767 TFs combination modes in the H1-hESc, GM12878 and K562 are shown in Figure 2B, 2D and 2F). The maximum PCC for combination modes of different amounts of HMs or different amounts of TFs is connected by a black curve. It is found that the predictive powers will basically reach summit in the maximum combination of four HMs or four TFs. The combination modes of maximum prediction accuracy for the four factors (i.e. four HMs or four TFs) are described in Table 2. These results show that all HMs+DNase or TFs are not equally important and there are statistical redundancies within HMs (TFs).

**Table 2:** The combination modes of the maximum prediction accuracy for four factors

cell line	factor	components for the combination	PCC
**H1**	TFs	POLR2A,SIX5,MAX,SUZ12	0.725
	HMs+DNase	H3K36me3, H3K27me3,H3K4me3,H3K9me3	0.763
**Gm12878**	TFs	GABPA,NFATC1,POLR2A,TCF3	0.789
	HMs+DNase	H3K79me2,H3K36me3,H3K27me3,H3K4me3	0.845
**K562**	TFs	ELF1,PML,POLR2A,ZBTB7A	0.791
	HMs+DNase	H3K36me3,H3K79me2,H3K9me3,H3K27me3	0.830

**Figure 3:**
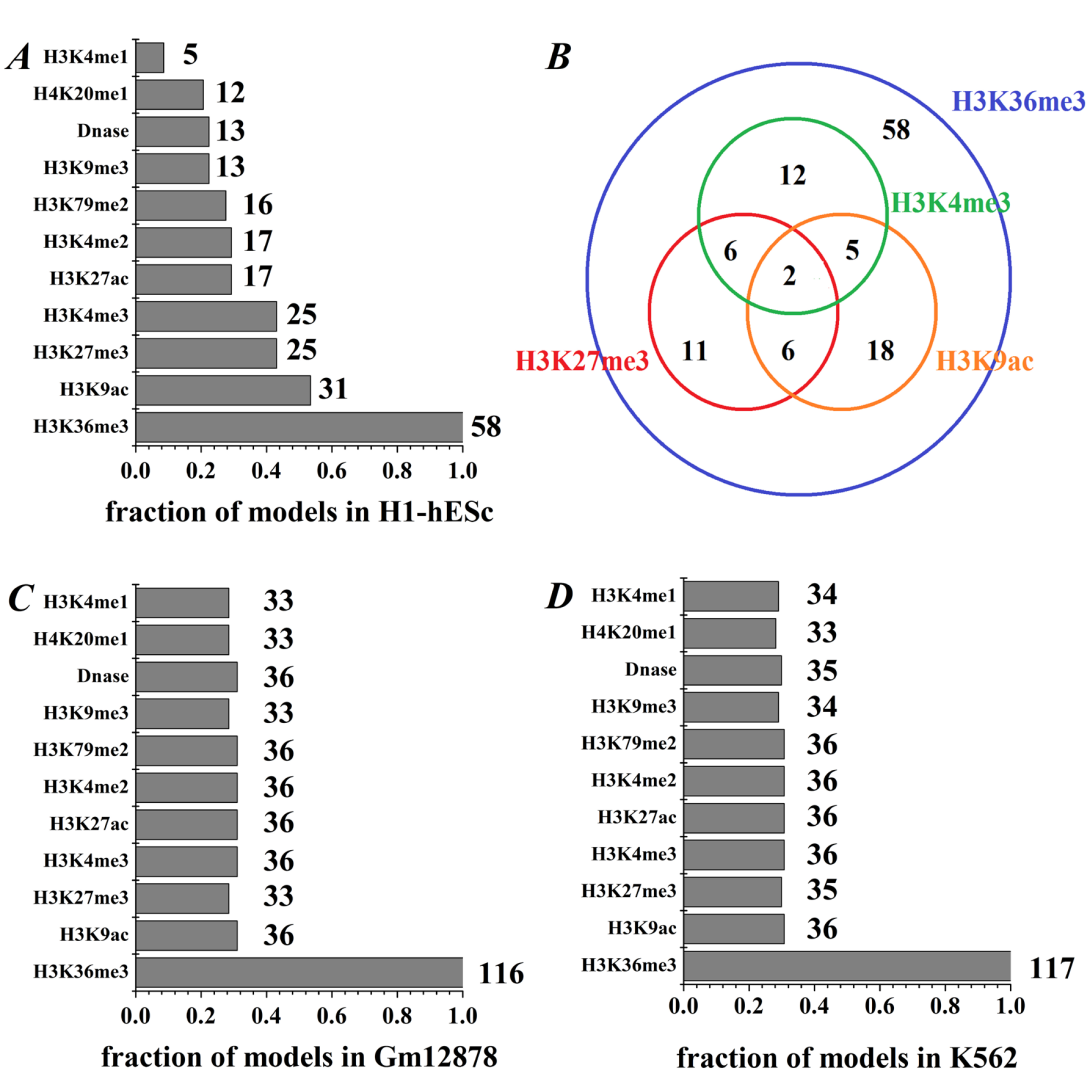
The appearance frequency of each HM in the studied modes **A**. The frequency of each HM in H1 cell line, where the integer represents the occurrence times in the studied modes. **B**. Venn diagram shows that the co-occurrence times of the four important HMs. **C**. and **D**. The frequency of each HM in Gm12878 and K562.

In addition, to further identify which HMs contribute more to predicting gene expression, we focus on the combinations modes of 4 kinds of HMs. We study all four-HMs modes whose PCC reach at least 95% of the all-HMs mode (PCC_all_H1_ = 0.786, PCC_all_Gm12878_ = 0.852 and PCC_all_K562_ = 0.836). There are finally 58, 116 and 117 combination modes, respectively, for H1-hESc, Gm12878 and K562, which is an enough large number to evaluate the over-representation analysis. By investigating the appearance times of each HM in these combination modes, we find the following results (see Figure 3):

Firstly, H3K36me3 appears in all these modes for the three cell lines, and it may be vital for gene expression. The better predictive results (PCC_H3K36me3_H1_ = 0.496, PCC_H3K36me3_Gm12878_ = 0.698 and PCC_H3K36me3_K562_ = 0.750) for gene expression levels are obtained by using single H3K36me3 information parameter. Our results are consistent with previous work, Hahn et al. showed H3K36me3 is a intragenic mark of active genes, and it is associated with two categories of genes [25]. Nanty et al. noticed that H3K36me3 has bimodalities in gene-body, which would influence DNA methylation levels and help shape gene-body CpG density profiles [26].

Secondly, for the H1-hESc, each of H3K9ac, H3K27me3 and H3K4me3 appears in nearly half of the 58 combination modes (53.45%, 43.10% and 43.10%, respectively), while other HMs appear in at most 29.31% of 58 modes (shown in Figure 3A). Thus, H3K9ac, H3K27me3 and H3K4me3 are more reliable information parameters than other HMs in H1-hESc, which consist with previous study [23]. Furthermore, we check the times that H3K9ac, H3K27me3, H3K4me3 and H3K36me3 appear together (shown in Figure 3B). We notice that H3K4me3 and H3K9ac appear simultaneously only seven times in the 58 modes, it may be that the information they represented are not simultaneously needed in 58 modes because their information redundancy, which is supported by the high correlation (PCC = 0.905). H3K4me3 and H3K27me3 (H3K27me3 and H3K9ac) occur together eight times in the 58 modes, and the correlation between H3K4me3 and H3K27me3 (H3K27me3 and H3K9ac) is PCC = 0.507 (PCC = 0.502), suggesting that they are partially redundant. However, we find H3K36me3 combines with one of H3K4me3, H3K27me3 and H3K9ac respectively appear in 23, 25 and 31 times, showing that the information they provide may be non-redundant. In fact, the correlations respectively are PCC = 0.097, PCC = 0.203 and PCC = 0.202.

Thirdly, for the Gm12878 and K562 cell lines, even though other HMs except H3K36me3 appear in similar level (about 30%, see Figure 3C and 3D), the combination of H3K36me3 and H3K79me2 can effectively increase the predictive power. We find the predictive accuracy of this combination in the four-HMs modes reaches at least 97.59% of the all-HMs mode.

Similarly, we focus on those four-TFs modes whose PCC reach at least 95% of the all-TFs mode (PCC_all_H1_ = 0.753, PCC_all_Gm12878_ = 0.799, PCC_all_K562_ = 0.802), 85, 172 and 345 modes are lastly remained for H1-hESc, Gm12878 and K562, respectively. We obtain that POLR2A is ubiquitous in all studied modes for the three cell lines and it can faithfully model gene expression levels (PCC_POLR2A_H1_ = 0.661, PCC_POLR2A_Gm12878_ = 0.677 and PCC_POLR2A_K562_ = 0.730). Previous researches had shown the importance of this mark which is linked to the synthesis of messenger RNA [27, 28]. For the K562 cell line, we also find the combination of POLR2A and ZBTB7A in the four-TFs modes reaches at least 97.58% of the all-TF mode. At last, to verify whether the above inferences depend on four-factors modes, we implement same analysis for five-factors and six-factors modes and analogous consequences are found.

### TFs and HMs provide similar effect on predicting genome-wide gene expression

As shown in Table 1, TFs and HMs model both obtain high predictive power, and TF+HM+DNase model only get similar predictive accuracy with them, indicating TFs binding and HMs may offer similar effects on genome-wide gene expression. To quantify this phenomenon, the PCC between the predictive values of TFs model and the predictive values of HMs model is respectively calculated for the three cell lines. Strong correlations (PCC_H1_ = 0.827, PCC_K562_ = 0.908, and PCC_Gm12878_ = 0.895 respectively) support that TFs and HMs offer similar effects on genome-wide gene expression and show the statistical redundancies also exist between TFs and HMs. Although TF+HM+DNase model does not obtain obviously improved predictive ability, it tends to more stable than TFs or HMs model (i.e. smaller *RMSE* between *R*^2^ and *CV-R*^2^ than TFs or HMs model).

### Regulation mechanism leads to statistical redundancy

**Figure 4:**
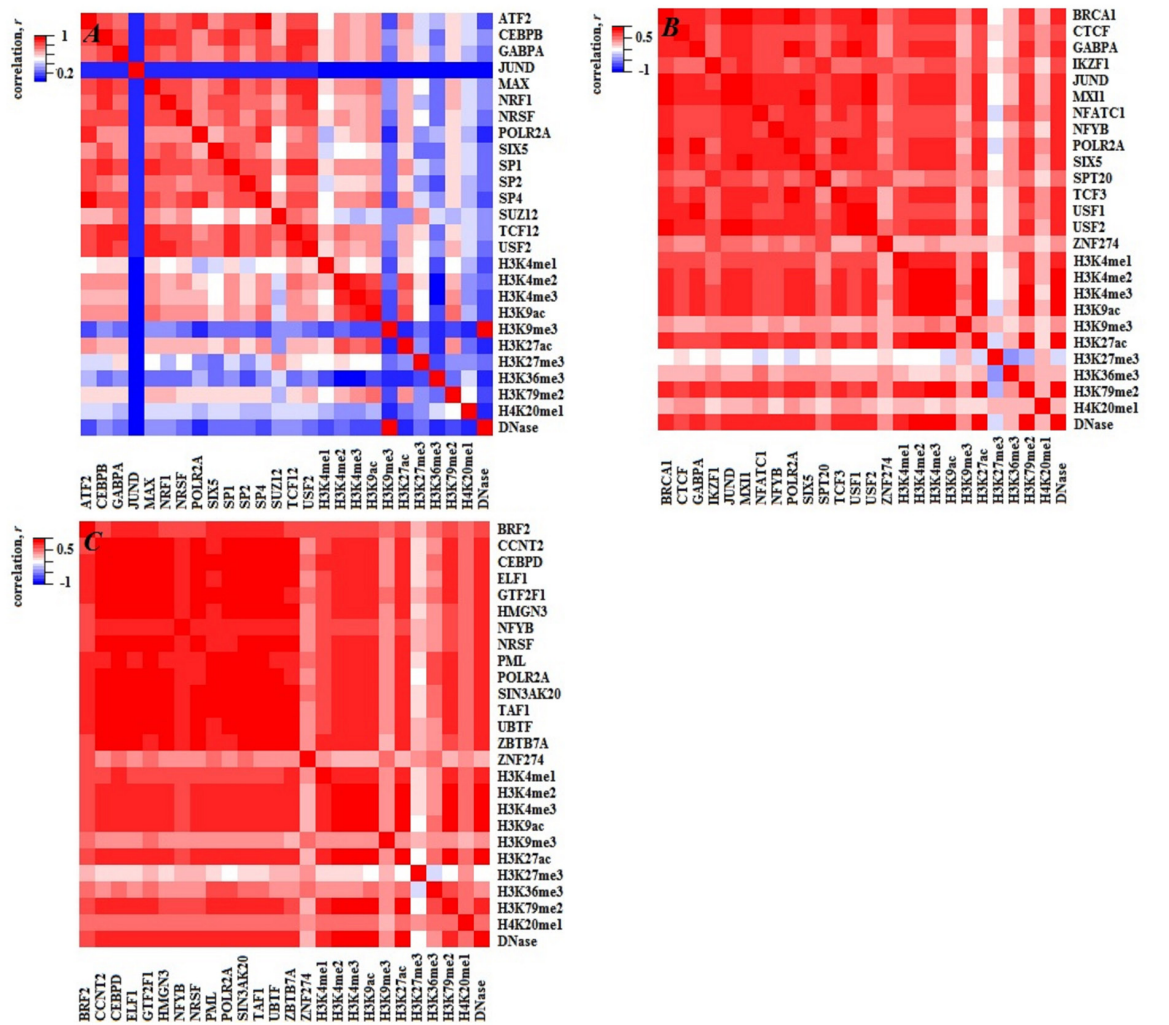
Heatmaps of PCC both within TFs (HMs) and between TFs and HMs for the three cell lines **A**., **B**. and **C**. represent H1, Gm12878 and K562 cell lines, respectively.

**Figure 5:**
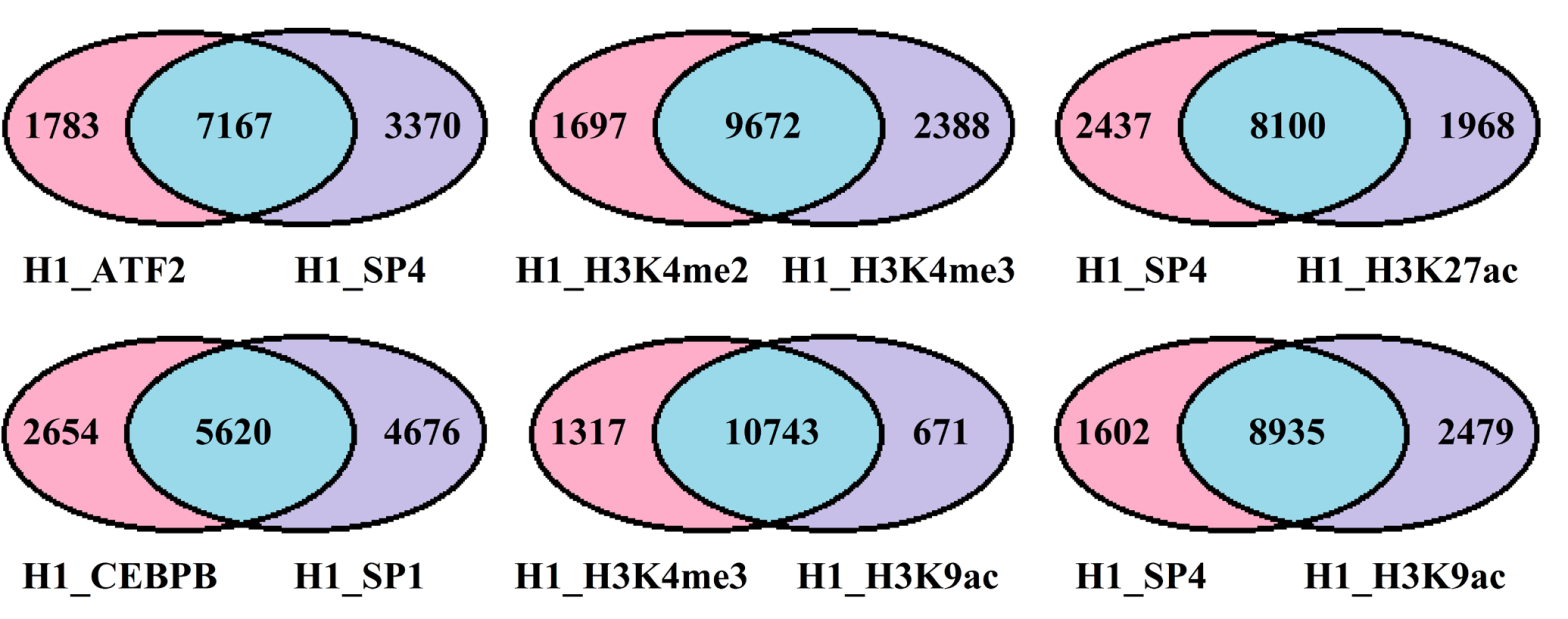
Venn diagram shows the number of the co-regulated and solo-regulated genes within and between TFs and HMs The blue depicts the co-regulated target genes, the pink and purple represent solo-regulated genes by factors attach to the charts, respectively.

To investigate the fundamental source of statistical redundancies among factors, the PCC between and within TFs and HMs are calculated for the three cell lines (see Figure 4). High correlations among these factors indicate the statistical redundancies maybe come from the regulation mechanism (i.e. two factors have similar regulatory functions). To verify the above supposition, the target genes of TFs or HMs are predicted by using the software BETA [29]. Then, the co-regulated and solo-regulated targets for TFs (HMs) whose PCC > 0.85 within TFs (HMs) and the co-regulated and solo-regulated targets for TF and HM whose PCC > 0.70 between TF and HM in H1-hESc cell lines are counted. The results present that the co-regulated genes are far more than solo-regulated genes for those factors (Figure 5 and Supplementary Figure S1, similar work is done for Gm12878 and K562 (not shown)), which effectively support our inferences. It is worth noting that some factors with similar regulatory functions have been demonstrated, for instance, CEBPB and SP1 which have strong correlation both can activate the expression of the insulin receptor gene [30]. Enrichments of H3K4me2 or H3K4me3 at TSS are positively correlated to the extents of gene activities [31], etc.

### Construction of TFs, HMs and gene expression interaction network

**Figure 6:**
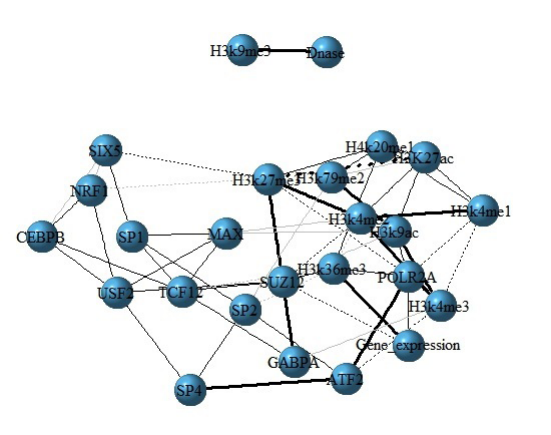
The interaction network among TFs, HMs and gene expression for H1 cell line In the network, nodes represent TFs, HMs and gene expression. Edges show the partial correlation coefficient between each paired factors, where the dash lines represent negative correlations and solid lines represent positive correlations. Bolder the line is, the stronger correlation it represents.

For further investigating how TFs, HMs interact with each other and the effects of TFs and HMs on gene expression, the interaction networks among TFs, HMs and gene expression are constructed, where Partial correlation coefficient is used to estimate inherent relationship between each paired factors and they are calculated as the edges of the networks. The entire process is done by R package ‘GeneNet_1.2.13’. Finally, 60 most significant edges are selected out for visualization (Figure 6 and Supplementary Figure S2).

For the three cell lines, we notice that H3K36me3 and POLR2A have direct correlations with gene expression levels and both promote the expression of genes, which maybe an interpretation why H3K36me3 and POLR2A are important in the section 2.3. Moreover, we find there is a higher positive correlation between H3K4me1 and H3K4me2 (between H3K4me2 and H3K4me3) in three cell lines. But the higher positive correlation between ATF2 and SP4 (between USF1 and USF2) is cell line specific. Besides, based on the interactive networks, we know that the gene expressions not only are affected by TFs and HMs, but also influenced by the interactions among factors (detailed in the legend of Figure 6). In order to check the robustness of the networks, we implement 50 times simulations by randomly removing 200 genes and same networks are found.

### The effects of TFs and HMs on prediction are different for genes in independent biological processes

In section 2.4, we find that TFs and HMs model offer similar predictive power for genome-wide gene expression. In order to further investigate the effects of TFs and HMs on prediction for genes in independent biological processes, we focus on the Gene Ontology biological processes [32, 33] for the high expression genes in the three cell lines (based on RPKM values, the top fifteen percent of all genes are selected as high expressed genes [3, 23]). Firstly, biological processes containing less than 30 genes are discarded, 1104, 1136 and 1070 sets of genes are remained, respectively, for H1-hESc, Gm12878 and K562 cell line. In order to ensure the effectiveness of statistics, the 604, 741 and 398 sets of genes for H1-hESc, Gm12878 and K562 cell line are lastly remained when TFs or HMs model's Benjamini-Hochberg-corrected *P*-value [34] is fewer than 0.05..

To quantify the effects of TFs and HMs on prediction for genes in independent biological processes, the ratio of PCC of TFs model to PCC of HMs model for these biological processes is calculated (see Supplementary tables 7-9 and Table 3). Of the 604, 741 and 398 biological processes for the three cell lines, it is found that 21, 89 and 24 processes show that the effect of HMs on prediction is superior to the effect of TFs (the ratio ranges from 0.59 to 0.90); 254, 235 and 65 processes show that the effect of TFs on prediction is superior to the effect of HMs (the ratio ranges from 1.10 to 2.01); but TFs and HMs offer similar effect on prediction in others 329, 418 and 309 processes (the ratio ranges from 0.90 to 1.10). In addition, we also notice that this phenomenon exists in same biology processes but in different cell lines (shown in Table 4). In conclusion, even though TFs and HMs offer similar effect on expression levels of genome-wide genes, the effects of TFs and HMs on predictive abilities are different for genes in some independent biological processes.

**Table 3:** List of three random GO-ID for each ratio range in the three cell lines

Cell lines	GO-ID	Go-term	TF_PCC	HM_PCC	Ratio
**H1**	GO:0010212	response to ionizing radiation	0.591	0.897	0.659
	GO:0046777	protein autophosphorylation	0.690	0.897	0.770
	GO:0016569	covalent chromatin modification	0.612	0.750	0.816
	GO:0006323	DNA packaging	0.775	0.834	0.929
	GO:0023061	signal release	0.890	0.926	0.961
	GO:0007409	axonogenesis	0.869	0.838	1.037
	GO:0007010	cytoskeleton organization	0.818	0.659	1.240
	GO:0006508	proteolysis	0.716	0.568	1.260
	GO:0030163	protein catabolic process	0.845	0.429	1.970
**Gm12878**	GO:0009117	nucleotide metabolic process	0.442	0.702	0.630
	GO:0040007	growth	0.630	0.869	0.725
	GO:0006875	cellular metal ion homeostasis	0.666	0.879	0.757
	GO:0065007	biological regulation	0.629	0.691	0.910
	GO:0016192	vesicle-mediated transport	0.781	0.805	0.970
	GO:0006325	chromatin organization	0.836	0.803	1.041
	GO:0045786	negative regulation of cell cycle	0.898	0.725	1.238
	GO:0006629	lipid metabolic process	0.853	0.654	1.304
	GO:0043087	regulation of GTPase activity	0.962	0.636	1.513
**K562**	GO:0023061	signal release	0.749	0.906	0.828
	GO:0007009	plasma membrane organization	0.820	0.933	0.879
	GO:0006396	RNA processing	0.583	0.651	0.894
	GO:0007155	cell adhesion	0.780	0.853	0.915
	GO:0030097	hemopoiesis	0.844	0.866	0.975
	GO:0030162	regulation of proteolysis	0.821	0.796	1.032
	GO:0006952	defense response	0.746	0.669	1.114
	GO:0045087	innate immune response	0.813	0.709	1.147
	GO:0051049	regulation of transport	0.807	0.608	1.329

**Table 4:** List of five random GO-ID where TFs and HMs model show distinct PCC for the same biological process in the different cell lines

GO-ID	GO_term	H1-TFs	H1-HMs	Gm12878 -TFs	Gm12878 -HMs	K562 -TFs	K562 -HMs
**GO:0042326**	negative regulation of phosphorylation	0.922	0.806	0.569	0.933	0.846	0.796
**GO:0009968**	negative regulation of signal transduction	0.767	0.689	0.651	0.860	0.738	0.723
**GO:0006873**	cellular ion homeostasis	0.942	0.866	0.668	0.897	0.693	0.815
**GO:0030003**	cellular cation homeostasis	0.939	0.874	0.668	0.897	0.692	0.830
**GO:0055080**	cation homeostasis	0.903	0.877	0.658	0.903	0.704	0.822

## DISCUSSION

The next-generation sequencing technology [35] provides large numbers of data that enable a more intensive research the interaction among TFs, HMs and DNA to be possible. Through a series of analyses and researches, the following interesting results can be put forward: (1) The selected TFs obtain better predictive than previous studies. Budden et al. [2] investigated the relation between core TFs and gene expression in Gm12878 by using similar method, their predictive accuracy was only CV-*R*^2^ = 0.390. But the predictive accuracy is CV-*R*^2^ = 0.617 in our study, this conclusion indicates that TFs studied in our paper may contain more information than those core TFs or can functionally substitute for some core TFs. The compared results are shown in Table 5. (2) Based on SVR model, the relationships between HMs and gene expression are investigated in Gm12878, and better results are obtained. For instance, McLeay et al. [36] studied the effects of 7 HMs and DNase on gene expression by a log-linear regression model, their predictive accuracy is CV-*R*^2^ = 0.412, but the predictive power in our study is CV-*R*^2^ = 0.719, which further imply a non-linear relations between HMs and gene expression. Dong et al. [24] constructed a two-step model to predict genes expression levels, they only use the chromatin feature density of ‘bestbin’ as predictor which ignores the information in other bins. Comparing with their accuracy PCC = 0.82, we achieve PCC = 0.85. The compared results are shown in Table 5. (3) In section 2.3 and 2.6, we and others observe that POLR2A, H3K4me3 and H3K27me3 can activate or inhibit gene expression [27, 28, 36–38], these not only show the obtained conclusions are accurate, but also indicate our model and methods may be reasonable.

**Table 5:** The predictive results compare with other studies

	cell lines	factors	CV-R^2^	method
**Budden’s study**	Gm12878	c-FOS,**CTCF**,EGR1,NRF1,NRSF,POU2F2, SP1,SRF,STAT3,**USF1**,YY1	0.390	SVR
**Our study**	Gm12878	**CTCF**,GABPA,IKZF1,JUND,MXI1,NFYB, NFATC1,SIX5,SPT20,TCF3,**USF1**,ZNF274, POLR2A,USF2,	0.617	SVR
**McLeay’s study**	Gm12878	**H3K4me1,H3K4me2,H4K20me1,H3K4me3**, **H3K36me3, H3K9me3,Dnase**	0.412	log-linear regression
**Our study**	Gm12878	H3K27ac,**H3K27me3**,H3K36me3,**H3K4me1**, **H3K4me2,H3K4me3**,H3K79me2,H3K9ac, **H3K9me3,H4K20me1,Dnase**	0.719	SVR
	**cell lines**	**factors**	**PCC**	**Method**
**Dong’s study**	H1 Gm12878 K562	H2AZ, **H3K27ac,H3K27me3,H3K36me3**, **H3K4me1,H3K4me2,H3K4me3,H3K79me2**, **H3K9ac,H3K9me3,H4K20me1,Dnase**	0.79 0.82 0.84	two-step
**Our study**	H1 Gm12878 K562	**H3K27ac,H3K27me3,H3K36me3,H3K4me1**, **H3K4me2,H3K4me3,H3K79me2,H3K9ac**, **H3K9me3,H4K20me1,Dnase**	0.79 0.85 0.84	SVR

The bold represents co-factors in the comparison.

Though improvements have been acquired, there are still some insufficiencies. In statistical prediction, the jackknife test is deemed the least arbitrary which had been elegantly demonstrated by Eqs. (28-30) in [39]. Hence, this method had been widely used by researchers to test the quality of information parameters (see, e.g., [40–46]). However, to reduce the computational time, the 10-fold cross validation is adopted in this paper as done by many researchers who use SVM as the prediction engine.

As future works, we will make our efforts to adopt more precise test method, and provide a publicly accessible and user-friendly web-server as presented in a series of recent publications [47–51] to effectively enhance their impacts [52]. Meanwhile, more precise and faster sequence analysis tools [53, 54] will be fully utilized in follow-on work.

## MATERIALS AND METHODS

### Available data and implementation

The RefSeq genes of the human genome (hg19) come from the UCSC database (http://genome.ucsc.edu/cgi-bin/hgTables), which contains transcription start site (TSS). Genes starting with NM are chosen out (i.e. the mature messenger RNA). In order to prevent the possibility that some genes may be the alternative transcripts of the same gene, only one of the genes which have the same TSS is retained. At last, a set of 19120 genes is left for remainder analysis.

All the TFs binding data, HMs profiles and DNase-I hypersensitivity data for H1-hESc, K562 and Gm12878cell lines are downloaded from the UCSC database (detail in Figure 1, Supplementary file Table S2 and Supplementary file Table S3). Because the DNase-I hypersensitivity data for the three cell lines are in hg18 coordinate, the UCSC liftOver tool [55] is used to convert the hg18 data into hg19. For visualization, the raw data is converted to bed format by using BEDtools software [56].

The expression data of the H1-hESc, Gm12878 and K562 are measured by applying the RNA-seq techniques. The mapped RNA-seq reads reported in this paper are depicted in the Gene Expression Omnibus database (GSM915329 (H1-hESc), GSM958730 (Gm12878) and GSM958731 (K562)). The expression levels of all genes are calculated according to the reads per kilobase of exon model per million mapped reads and represented as RPKM value [57].

### Transcription factors binding signal

The DNA regions flanking the TSS (-10~10kb) of all RefSeq genes are separated into 100 bins, each of 200 bps in size. Based on our previous study [3], signals of TFs binding are normalized by using the following Eq. (1),

$$N_{ij}^{k}=(n_{i,j}^{k}\times 10^{9})/(n_{tag}^{k}\times 200)$$

 (1)

in which *N*^k^_ij_ represents normalized signal, *n*^k^_ij_ is the total tags that *k*-*th* TF locates in the *j*-th bin of the *i*-th gene, 10^9^ is used to eliminate the difference of magnitude with RPKM. 200 is the length of the *j*-th bin, and *n*^k^_tag_ is the total tags of the *k*-*th* TF. This results in a 19120×100 matrix *N* (matrix element is *N*^k^_ij_ (*i* = 1, 2,…,19120; *j* = 1,2,…,100; *k* = 1,2,…,15) for the *k*-th TF.

### HMs and DNase binding signal

Similarly, the DNA regions flanking the TSS (-2~2kb) of all RefSeq genes are divided into 20 bins, with each consisting of 200 bps. Then, the signals of HMs and DNase binding are normalized by using the following Eq. (2),

$$H_{im}^{l}=(h_{i,m}^{l}\times 10^{9})/(h_{tag}^{l}\times 200)$$

 (2)

where *H*^l^_im_ represents the normalized signal, *h*^l^_im_ is the total tags that *l*-th HM or DNase locates in the *m*-th bin of the *i*-th gene, and *h*^l^_tag_ is the total tags of the *l*-*th* HM or DNase. This results in a 19120×20 matrix *H* (matrix element is *H*^l^_im_ (*m* = 1,2,…,20; *l* = 1,2,…,11) for the *l*-th HM or DNase.

### Calculation of TFs association strength (TFAS)

For the *i*-th gene and the *k*-th TF, TFAS is calculated by the following Eq. (3)

$$a_{ik}=\sum_{j=1}^{100}N_{ij}^{k}F_{k}(d_{j})$$

$$a_{ik}^{'}=\log_{2}(a_{ik}+\sigma_{k})$$

 (3)

where *N*^k^_ij_ is computed by Eq.(1), *F*k is the normalized Gaussian kernel density function, where the bandwidth is calculated by the rule of thumb [58]. *d*j is a relative distance between the midpoint of the *j-*th bin and the corresponding gene's TSS, the *σ*_k_ is a pseudocount (the detailed information is displayed in supplementary information). For 19120 genes and 15 TFs, the TFAS profiles are denoted by the 19120×15 matrix *a* (the matrix element is^*a_ik_*^).

### Calculation of HMs or DNase association strength (HMAS)

For the *i*-th gene and the *l*-th HM or DNase, the HMAS is calculated by using the following Eq. (4)

$$b_{il}=\sum_{m=1}^{20}H_{im}^{l}$$

$$b_{il}^{'}=\log_{2}(b_{il}+\sigma_{l})$$

 (4)

where *H*^l^_im_ is computed by Eq.(2), the *σ*_i_ is a pseudocount, the HMAS profiles are denoted by the 19120×11 matrix *b* (the matrix element is^*b_il_*^).

### Log-linear regression model and non-linear SVR model

Combining with the TFASs, HMASs and multivariate linear regression, the log-linear regression model is derived by the following Eq. (5)

$$\log_{2}(L_{i}+\sigma )=\nu +\sum_{k=1}^{15}\alpha_{k}a_{ik}^{'}+\sum_{l=1}^{11}\beta_{l}b_{il}^{'}$$

 (5)

in which *L*i is the RPKM value of the *i*-th gene, σ is a pseudocount, *υ* is the intercept, *α*k and *β*l are the regression coefficients.

Based on the support vector machines, a SVR model is constructed by using the Eq. (6)

$$\log_{2}(L_{i}+\sigma )=\mu +\sum_{i}\gamma_{i}\cdot K(X_{i},\;X)$$

 (6)

in which *μ* is the intercept, *K*(*X*i, *X*) is the kernel function and *γ*_i_ is the Lagrange multiplier. Matrix *X* is the matrix a (calculated by Eq.(3)) and/or the matrix b (calculated by Eq.(4)), *X*i is the *i*-th row elements of matrix X. The entire process is done by libSVM software [59].

## SUPPLEMENTARY MATERIALS FIGURES AND TABLES























## Abbreviations

TF: transcription factor
HM: histone modification
SVR: support vector regression
PCC: Pearson correlation coefficient
TSS: transcription start site
RPKM: reads per kilobase of exon model per million mapped reads
TFAS: Transcription factors association strength
HMAS: histone modifications or DNase association strength;.
