# Supplementary material for: Estimating the effects of transcription factors binding and histone modifications on gene expression levels in human cells
Source: Oncotarget. 2017 Apr 9;8(25):40090–103. doi: 10.18632/oncotarget.16988 (PMC5522221; doi:10.18632/oncotarget.16988)
Supplement: Supplementary file 1 [file oncotarget-08-40090-s001.pdf]

Estimating the effects of transcription factors binding and histone modifications on gene expression levels in human cells

Supplementary Material

Supplementary Information

1. The selection of transcription factors for the three cell lines

Previous studies [1, 2] have shown a small panel of transcription factors (TFs) can be used to predict gene expression with a high degree of accuracy. In this study, we use stepwise regression analysis [2] to select important TFs for predicting gene expression from the hundreds of TFs in the three cell lines. The stepwise regression analysis firstly diagnoses the TF which has the strongest prediction power for gene expression levels. Next, the variables are added or removed one by one to achieve better prediction accuracies and statistical significances. Finally, the top 15 TFs for each cell line are chosen out and regarded as "optimal" TFs to predict gene expression levels.

2. Estimating the pseudocount σ

Because the a_{ik} and b_{il} are subsequently transformed to a logarithmic scale, pseudocount σ will be added to each a_{ik} or b_{il} to avoid undefined values of the logarithm when a_{ik} and b_{il} equals zero. For each TF or HM, the search space for σ ranges from 10^{-10} to 10^{10}. Each σ is used to transform a_{ik} and b_{il} into a'_{ik} and b'_{il} and the σ which maximizes the correlation between a'_{ik} (b'_{il}) and the logarithm of measured expression values is chosen for further analyses.

REFERENCES

1. Vaquerizas JM, Kummerfeld SK, Teichmann SA, Luscombe NM. A census of human transcription factors: function, expression and evolution. Nature Reviews Genetics. 2009; 10:252-263.

2. Hwang JS, Hu TH. A stepwise regression algorithm for high-dimensional variable selection. J Stat Comput Simul. 2015; 85:1793-1806.

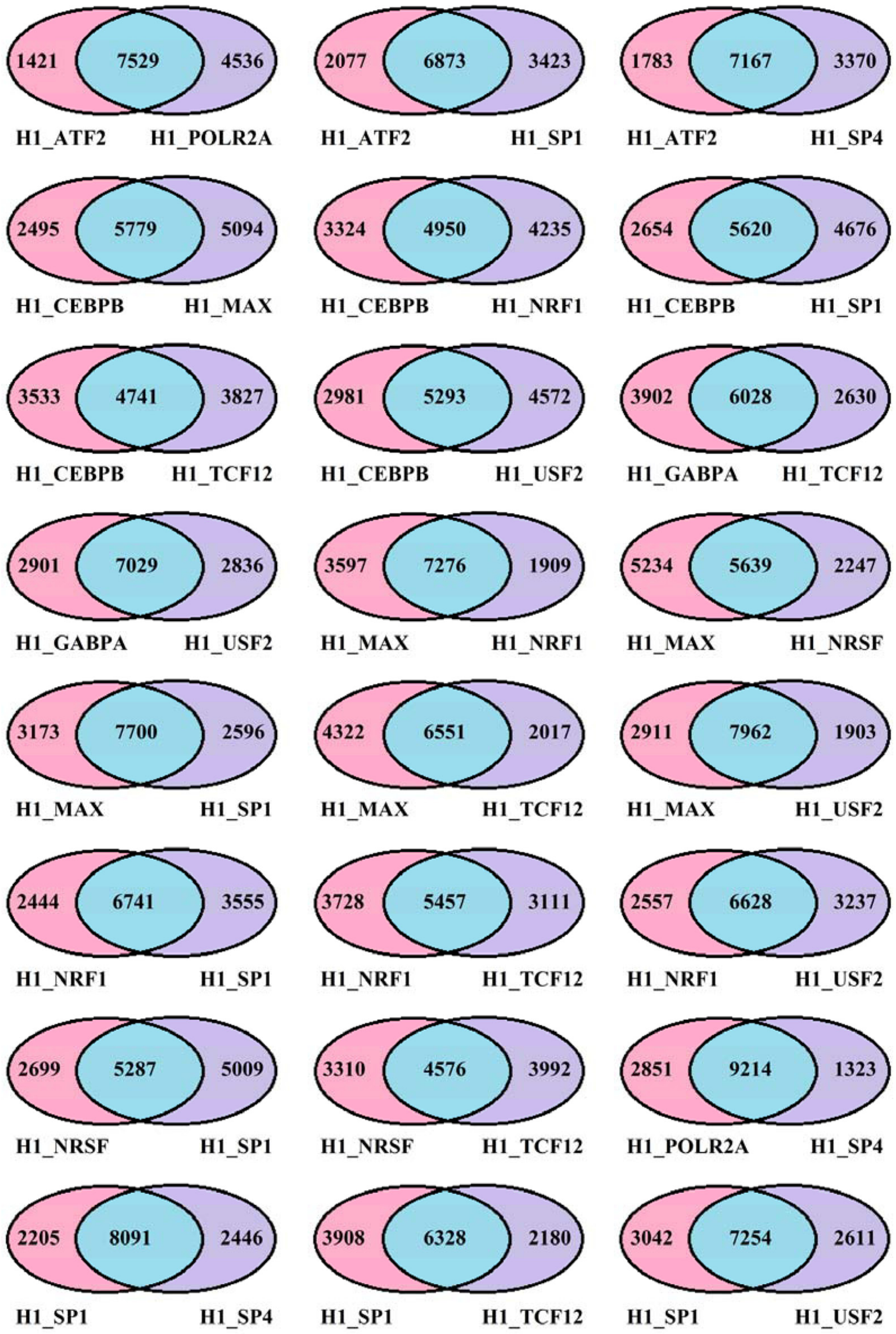
1421
7529
4536
H1_ATF2
H1_POLR2A
2077
6873
3423
H1_ATF2
H1_SP1
1783
7167
3370
H1_ATF2
H1_SP4
2495
5779
5094
H1_CEBPB
H1_MAX
3324
4950
4235
H1_CEBPB
H1_NRF1
2654
5620
4676
H1_CEBPB
H1_SP1
3533
4741
3827
H1_CEBPB
H1_TCF12
2981
5293
4572
H1_CEBPB
H1_USF2
3902
6028
2630
H1_GABPA
H1_TCF12
2901
7029
2836
H1_GABPA
H1_USF2
3597
7276
1909
H1_MAX
H1_NRF1
5234
5639
2247
H1_MAX
H1_NRSF
3173
7700
2596
H1_MAX
H1_SP1
4322
6551
2017
H1_MAX
H1_TCF12
2911
7962
1903
H1_MAX
H1_USF2
2444
6741
3555
H1_NRF1
H1_SP1
3728
5457
3111
H1_NRF1
H1_TCF12
2557
6628
3237
H1_NRF1
H1_USF2
2699
5287
5009
H1_NRSF
H1_SP1
3310
4576
3992
H1_NRSF
H1_TCF12
2851
9214
1323
H1_POLR2A
H1_SP4
2205
8091
2446
H1_SP1
H1_SP4
3908
6328
2180
H1_SP1
H1_TCF12
3042
7254
2611
H1_SP1
H1_USF2

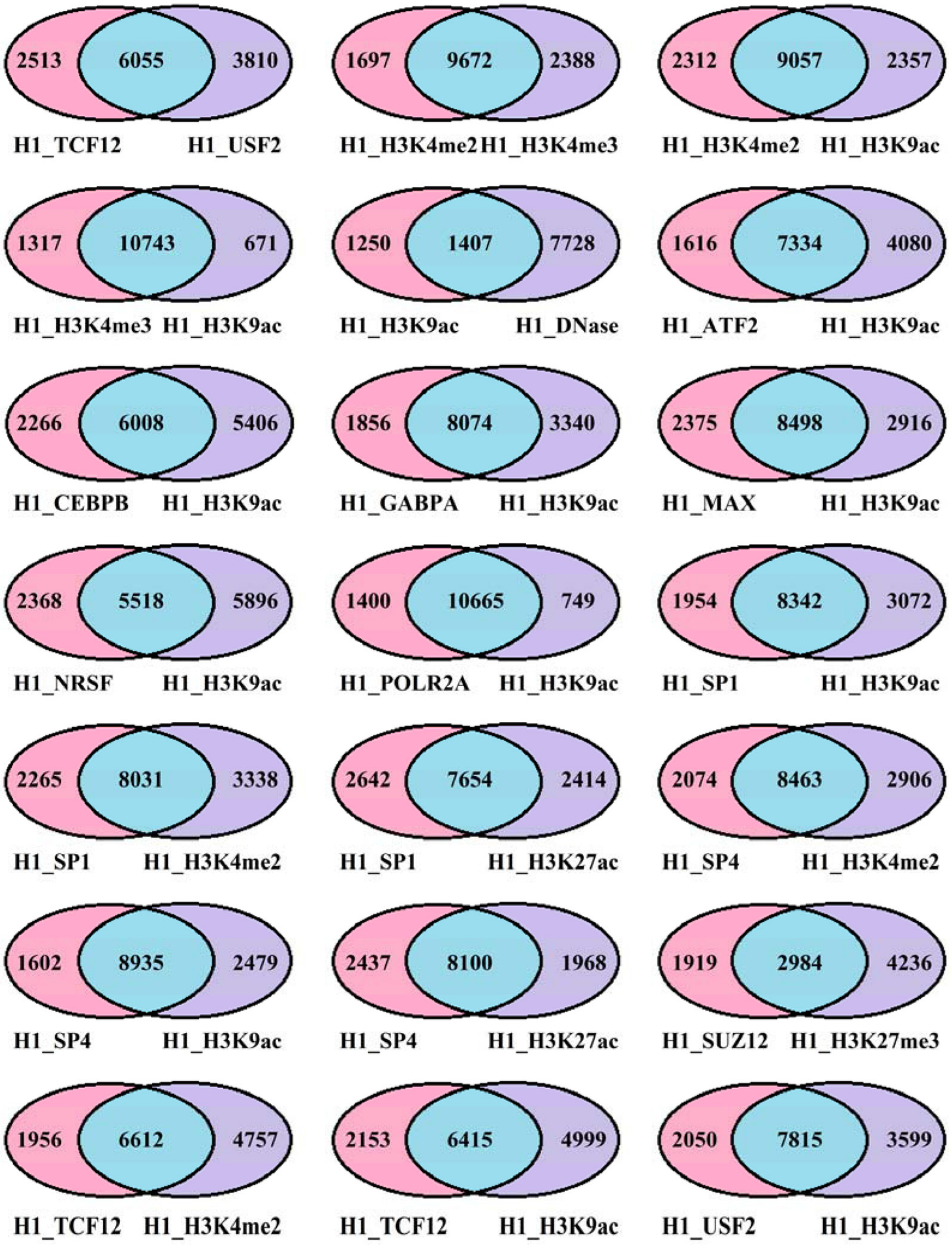

Supplementary Figure S1: Venn diagram shows the number of the co-regulated target genes and solo-regulated genes within and between TFs and HMs. The blue depicts the co-regulated target genes, the pink and purple respectively represent solo-regulated genes by factors attach to the charts.

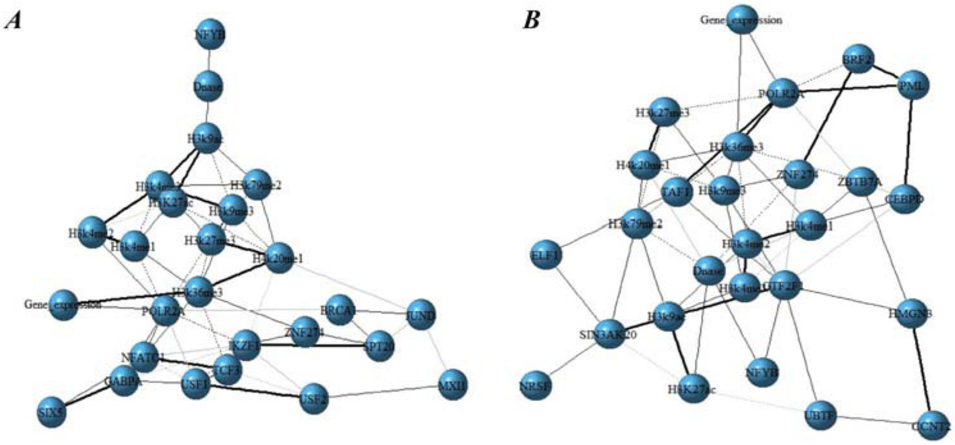

Supplementary Figure S2: The interaction network among TFs, HMs and gene expression for Gm12878 and K562. (A) Gm12878 and (B) K562 cell line. In the network, nodes represent TFs, HMs and gene expression. Edges show the partial correlation coefficient between each paired vectors, where the dash lines represent negative correlations and solid lines represent positive correlations. Bolder the line is, the stronger correlation it represents.

Supplementary File 1: Detailed method for seleting TFs and calculating pseudocount σ, Table S1-S3 and Figure S1-S2 with their legends.
Supplementary Table 1: 32767 TFs combination modes and their predictive abilities in H1 cell line.
Supplementary Table 2: 32767 TFs combination modes and their predictive abilities in Gm12878 cell line.
Supplementary Table 3: 32767 TFs combination modes and their predictive abilities in K562 cell line.
Supplementary Table 4: 2047 HMs combination modes and their predictive abilities in H1 cell line.
Supplementary Table 5: 2047 HMs combination modes and their predictive abilities in Gm12878 cell line.
Supplementary Table 6: 2047 HMs combination modes and their predictive abilities in K562 cell line.
Supplementary Table 7: The predictive results of TFs and HMs for the 604 biological processes in H1 cell line.
Supplementary Table 8: The predictive results of TFs and HMs for the 741 biological processes in Gm12878 cell line.
Supplementary Table 9: The predictive results of TFs and HMs for the 398 biological processes in K562 cell line.

For Supplementary Files see in Supplementary Files
