# Supplementary material for: Estimating the effects of transcription factors binding and histone modifications on gene expression levels in human cells
Source: Oncotarget. 2017 Apr 9;8(25):40090–103. doi: 10.18632/oncotarget.16988 (PMC5522221; doi:10.18632/oncotarget.16988)
Supplement: Supplementary file 2 [file oncotarget-08-40090-s002.doc]

Supplementary Table 1. The prediction accuracy of the 10-fold cross-validation in the three cell lines. The *CV*- *R*^2^ is the average *R*^2^ for the 10 fold cross-validation.

|  | **n** | **TF(log-line)** | **TF(SVR)** | **HMs+Dnase**  **(log-line)** | **HMs+Dnase**  **(SVR)** | **TF+ HMs+**  **Dnase(log-line)** | **TF+HMs+**  **Dnase(SVR)** |
| --- | --- | --- | --- | --- | --- | --- | --- |
| **H1** | 1 | 0.402 | 0.553 | 0.532 | 0.608 | 0.566 | 0.649 |
|  | 2 | 0.392 | 0.536 | 0.510 | 0.569 | 0.542 | 0.616 |
|  | 3 | 0.405 | 0.549 | 0.535 | 0.601 | 0.558 | 0.640 |
|  | 4 | 0.384 | 0.531 | 0.534 | 0.610 | 0.557 | 0.642 |
|  | 5 | 0.428 | 0.534 | 0.525 | 0.583 | 0.557 | 0.630 |
|  | 6 | 0.400 | 0.531 | 0.548 | 0.606 | 0.558 | 0.635 |
|  | 7 | 0.412 | 0.537 | 0.507 | 0.587 | 0.545 | 0.624 |
|  | 8 | 0.409 | 0.566 | 0.534 | 0.589 | 0.570 | 0.639 |
|  | 9 | 0.394 | 0.552 | 0.523 | 0.595 | 0.545 | 0.644 |
|  | 10 | 0.416 | 0.550 | 0.537 | 0.587 | 0.550 | 0.630 |
|  | *CV-R*^2^ | 0.404 | 0.544 | 0.529 | 0.594 | 0.555 | 0.635 |
| **Gm12878** | 1 | 0.482 | 0.610 | 0.646 | 0.710 | 0.676 | 0.724 |
|  | 2 | 0.515 | 0.612 | 0.674 | 0.714 | 0.631 | 0.724 |
|  | 3 | 0.512 | 0.632 | 0.670 | 0.709 | 0.650 | 0.729 |
|  | 4 | 0.500 | 0.630 | 0.673 | 0.724 | 0.658 | 0.734 |
|  | 5 | 0.486 | 0.617 | 0.669 | 0.726 | 0.650 | 0.735 |
|  | 6 | 0.518 | 0.641 | 0.690 | 0.741 | 0.639 | 0.753 |
|  | 7 | 0.502 | 0.608 | 0.661 | 0.711 | 0.651 | 0.726 |
|  | 8 | 0.471 | 0.593 | 0.652 | 0.709 | 0.632 | 0.711 |
|  | 9 | 0.488 | 0.624 | 0.681 | 0.736 | 0.656 | 0.746 |
|  | 10 | 0.473 | 0.600 | 0.668 | 0.712 | 0.648 | 0.720 |
|  | *CV*-*R*^2^ | 0.495 | 0.617 | 0.668 | 0.719 | 0.649 | 0.730 |
| **K562** | 1 | 0.546 | 0.634 | 0.666 | 0.707 | 0.651 | 0.697 |
|  | 2 | 0.534 | 0.641 | 0.661 | 0.715 | 0.650 | 0.708 |
|  | 3 | 0.489 | 0.597 | 0.616 | 0.663 | 0.601 | 0.654 |
|  | 4 | 0.539 | 0.646 | 0.661 | 0.703 | 0.647 | 0.704 |
|  | 5 | 0.549 | 0.634 | 0.635 | 0.677 | 0.634 | 0.686 |
|  | 6 | 0.544 | 0.654 | 0.630 | 0.681 | 0.640 | 0.696 |
|  | 7 | 0.534 | 0.617 | 0.638 | 0.684 | 0.629 | 0.684 |
|  | 8 | 0.491 | 0.602 | 0.619 | 0.674 | 0.608 | 0.667 |
|  | 9 | 0.521 | 0.626 | 0.638 | 0.698 | 0.634 | 0.694 |
|  | 10 | 0.523 | 0.617 | 0.648 | 0.696 | 0.639 | 0.692 |
|  | *CV*-*R*^2^ | 0.527 | 0.627 | 0.641 | 0.690 | 0.633 | 0.688 |

Supplementary Table S2: List of the 10 HMs and Dnase involved in the current study for the corresponding cell line.

| **cell line** | **Number of HMs and Dnase** | **List of TFs in the corresponding cell** |
| --- | --- | --- |
| **H1**  **Human embryonic stem cells, line H1** | 11 | H3K27ac,H3K27me3,H3K36me3,H3K4me1,  H3K4me2,H3K4me3,H3K79me2,H3K9ac,  H3K9me3,H4K20me1,Dnase |
| **GM12878**  **B-lymphoblastoid**  **cell line** | 11 | H3K27ac,H3K27me3,H3K36me3,H3K4me1,  H3K4me2,H3K4me3,H3K79me2,H3K9ac,  H3K9me3,H4K20me1,Dnase |
| **K562**  **Chronic myelogenous/ erythroleukemia cell line** | 11 | H3K27ac,H3K27me3,H3K36me3,H3K4me1,  H3K4me2,H3K4me3,H3K79me2,H3K9ac,  H3K9me3,H4K20me1,Dnase |

Supplementary Table 3: List of datasets used in this study. All data files can be downloaded from the stated sub-directories of the following URL: http://genome.ucsc.edu/ENCODE/downloads.html

| Transcription factor | Sub-directory | Dataset ID | Version |
| --- | --- | --- | --- |
| ATF2 | wgEncodeHaibTfbs | wgEncodeHaibTfbsH1hescAtf2 | Hg19 |
| CEBPB | wgEncodeSydhTfbs | wgEncodeSydhTfbsH1hescCebpb | Hg19 |
| GABPA | wgEncodeHaibTfbs | [wgEncodeHaibTfbsH1hescGabp](http://hgdownload.cse.ucsc.edu/goldenPath/hg19/encodeDCC/wgEncodeHaibTfbs/wgEncodeHaibTfbsH1hescGabpPcr1xAlnRep1.bam) | Hg19 |
| JUND | wgEncodeHaibTfbs | wgEncodeHaibTfbsH1hescJund | Hg19 |
| MAX | wgEncodeHaibTfbs | wgEncodeHaibTfbsH1hescMax | Hg19 |
| NRF1 | wgEncodeSydhTfbs | wgEncodeSydhTfbsH1hescNrf1 | Hg19 |
| NRSF | wgEncodeHaibTfbs | wgEncodeHaibTfbsH1hescNrsf | Hg19 |
| POLR2A | wgEncodeHaibTfbs | wgEncodeSydhTfbsH1hescPolr | Hg19 |
| SIX5 | wgEncodeHaibTfbs | wgEncodeHaibTfbsH1hescSix5 | Hg19 |
| SP1 | wgEncodeHaibTfbs | wgEncodeHaibTfbsH1hescSp1 | Hg19 |
| SP2 | wgEncodeHaibTfbs | wgEncodeHaibTfbsH1hescSp2 | Hg19 |
| SP4 | wgEncodeHaibTfbs | wgEncodeHaibTfbsH1hescSp4 | Hg19 |
| SUZ12 | wgEncodeSydhTfbs | wgEncodeSydhTfbsH1hescSuz12 | Hg19 |
| TCF12 | wgEncodeHaibTfbs | wgEncodeHaibTfbsH1hescTcf12 | Hg19 |
| USF2 | wgEncodeSydhTfbs | wgEncodeSydhTfbsH1hescUsf2 | Hg19 |
| H3K27ac | wgEncodeBroadHistone | wgEncodeBroadHistoneH1hescH3k27ac | Hg19 |
| H3K27me3 | wgEncodeBroadHistone | wgEncodeBroadHistoneH1hescH3k27me3 | Hg19 |
| H3K36me3 | wgEncodeBroadHistone | wgEncodeBroadHistoneH1hescH3k36me3 | Hg19 |
| H3K4me1 | wgEncodeBroadHistone | wgEncodeBroadHistoneH1hescH3k4me1 | Hg19 |
| H3K4me2 | wgEncodeBroadHistone | wgEncodeBroadHistoneH1hescH3k4me2 | Hg19 |
| H3K4me3 | wgEncodeBroadHistone | wgEncodeBroadHistoneH1hescH3k4me3 | Hg19 |
| H3K79me2 | wgEncodeBroadHistone | wgEncodeBroadHistoneH1hescH3k79me2 | Hg19 |
| H3K9ac | wgEncodeBroadHistone | wgEncodeBroadHistoneH1hescH3k9ac | Hg19 |
| H3K9me3 | wgEncodeBroadHistone | wgEncodeBroadHistoneH1hescH3k9me3 | Hg19 |
| H4K20me1 | wgEncodeBroadHistone | wgEncodeBroadHistoneH1hescH4k20me1 | Hg19 |
| Dnase | wgEncodeUwDnase | wgEncodeUwDnaseH1hesc | Hg18 |
| CTCF | wgEncodeSydhTfbs | wgEncodeSydhTfbsGm12878Ctcf | Hg19 |
| BRCA1 | wgEncodeSydhTfbs | wgEncodeSydhTfbsGm12878Brca1 | Hg19 |
| GABPA | wgEncodeHaibTfbs | wgEncodeHaibTfbsGm12878Gabp | Hg19 |
| IKZF1 | wgEncodeSydhTfbs | wgEncodeSydhTfbsGm12878Ikzf1 | Hg19 |
| JUND | wgEncodeSydhTfbs | wgEncodeSydhTfbsGm12878Jund | Hg19 |
| MXI1 | wgEncodeSydhTfbs | wgEncodeSydhTfbsGm12878Mxi1 | Hg19 |
| NFATC1 | wgEncodeHaibTfbs | wgEncodeHaibTfbsGm12878Nfatc1 | Hg19 |
| NFYB | wgEncodeSydhTfbs | wgEncodeSydhTfbsGm12878Nfyb | Hg19 |
| POLR2A | wgEncodeHaibTfbs | wgEncodeHaibTfbsGm12878Pol2 | Hg19 |
| SIX5 | wgEncodeHaibTfbs | wgEncodeHaibTfbsGm12878Six5 | Hg19 |
| SPT20 | wgEncodeSydhTfbs | wgEncodeSydhTfbsGm12878Spt20 | Hg19 |
| TCF3 | wgEncodeHaibTfbs | wgEncodeHaibTfbsGm12878Tcf3 | Hg19 |
| USF1 | wgEncodeHaibTfbs | wgEncodeHaibTfbsGm12878Usf1 | Hg19 |
| USF2 | wgEncodeSydhTfbs | wgEncodeSydhTfbsGm12878Usf2 | Hg19 |
| ZNF274 | wgEncodeSydhTfbs | wgEncodeSydhTfbsGm12878Znf274 | Hg19 |
| H3K27ac | wgEncodeBroadHistone | wgEncodeBroadHistoneGm12878H3k27ac | Hg19 |
| H3K27me3 | wgEncodeBroadHistone | wgEncodeBroadHistoneGm12878H3k27me3 | Hg19 |
| H3K36me3 | wgEncodeBroadHistone | wgEncodeBroadHistoneGm12878H3k36me3 | Hg19 |
| H3K4me1 | wgEncodeBroadHistone | wgEncodeBroadHistoneGm12878H3k4me1 | Hg19 |
| H3K4me2 | wgEncodeBroadHistone | wgEncodeBroadHistoneGm12878H3k4me2 | Hg19 |
| H3K4me3 | wgEncodeBroadHistone | wgEncodeBroadHistoneGm12878H3k4me3 | Hg19 |
| H3K79me2 | wgEncodeBroadHistone | wgEncodeBroadHistoneGm12878H3k79me2 | Hg19 |
| H3K9ac | wgEncodeBroadHistone | wgEncodeBroadHistoneGm12878H3k9ac | Hg19 |
| H3K9me3 | wgEncodeBroadHistone | wgEncodeBroadHistoneGm12878H3k9me3 | Hg19 |
| H4K20me1 | wgEncodeBroadHistone | wgEncodeBroadHistoneGm12878H4k20me1 | Hg19 |
| Dnase | wgEncodeUwDnase | wgEncodeUwDnaseGm12878 | Hg18 |
| BRF2 | wgEncodeSydhTfbs | wgEncodeSydhTfbsK562Brf2 | Hg19 |
| CCNT2 | wgEncodeSydhTfbs | wgEncodeSydhTfbsK562Ccnt2 | Hg19 |
| CEBPD | wgEncodeHaibTfbs | wgEncodeHaibTfbsK562Cebpd | Hg19 |
| ELF1 | wgEncodeHaibTfbs | wgEncodeHaibTfbsK562Elf1 | Hg19 |
| GTF2F1 | wgEncodeSydhTfbs | wgEncodeSydhTfbsK562Gtf2 | Hg19 |
| HMGN3 | wgEncodeSydhTfbs | wgEncodeSydhTfbsK562Hmgn3 | Hg19 |
| NFYB | wgEncodeSydhTfbs | wgEncodeSydhTfbsK562Nfyb | Hg19 |
| NRSF | wgEncodeHaibTfbs | wgEncodeHaibTfbsK562Nrsf | Hg19 |
| PML | wgEncodeHaibTfbs | wgEncodeHaibTfbsK562Pml | Hg19 |
| POLR2A | wgEncodeSydhTfbs | wgEncodeSydhTfbsK562Pol2 | Hg19 |
| SIN3AK20 | wgEncodeHaibTfbs | wgEncodeHaibTfbsK562Sin3ak20 | Hg19 |
| TAF1 | wgEncodeHaibTfbs | wgEncodeHaibTfbsK562Taf1 | Hg19 |
| UBTF | wgEncodeSydhTfbs | wgEncodeSydhTfbsK562Ubtf | Hg19 |
| ZNF274 | wgEncodeSydhTfbs | wgEncodeSydhTfbsK562Znf274 | Hg19 |
| ZBTB7A | wgEncodeHaibTfbs | wgEncodeHaibTfbsK562Zbtb7a | Hg19 |
| H3K27ac | wgEncodeBroadHistone | wgEncodeBroadHistoneK562H3k27ac | Hg19 |
| H3K27me3 | wgEncodeBroadHistone | wgEncodeBroadHistoneK562H3k27me3 | Hg19 |
| H3K36me3 | wgEncodeBroadHistone | wgEncodeBroadHistoneK562H3k36me3 | Hg19 |
| H3K4me1 | wgEncodeBroadHistone | wgEncodeBroadHistoneK562H3k4me1 | Hg19 |
| H3K4me2 | wgEncodeBroadHistone | wgEncodeBroadHistoneK562H3k4me2 | Hg19 |
| H3K4me3 | wgEncodeBroadHistone | wgEncodeBroadHistoneK562H3k4me3 | Hg19 |
| H3K79me2 | wgEncodeBroadHistone | wgEncodeBroadHistoneK562H3k79me2 | Hg19 |
| H3K9ac | wgEncodeBroadHistone | wgEncodeBroadHistoneK562H3k9ac | Hg19 |
| H3K9me3 | wgEncodeBroadHistone | wgEncodeBroadHistoneK562H3k9me3 | Hg19 |
| H4K20me1 | wgEncodeBroadHistone | wgEncodeBroadHistoneK562H4k20me1 | Hg19 |
| Dnase | wgEncodeUwDnase | wgEncodeUwDnaseK562 | Hg18 |
